# Generating Instructive Questions from Multiple Articles to Guide Reading in E-Bibliotherapy

**DOI:** 10.3390/s21093223

**Published:** 2021-05-06

**Authors:** Yunxing Xin, Lei Cao, Xin Wang, Xiaohao He, Ling Feng

**Affiliations:** Centre for Computational Mental Healthcare, Department of Computer Science and Technology, Research Institute of Data Science, Tsinghua University, Beijing 100084, China; xinyx16@mails.tsinghua.edu.cn (Y.X.); cao-l17@mails.tsinghua.edu.cn (L.C.); xin-wang18@mails.tsinghua.edu.cn (X.W.); hexh17@mails.tsinghua.edu.cn (X.H.)

**Keywords:** E-bibliotherapy, instructive question, reading guidance, encoder-decoder, dataset

## Abstract

E-Bibliotherapy deals with adolescent psychological stress by manually or automatically recommending multiple reading articles around their stressful events, using electronic devices as a medium. To make E-Bibliotherapy really useful, generating instructive questions before their reading is an important step. Such a question shall (a) attract teens’ attention; (b) convey the essential message of the reading materials so as to improve teens’ active comprehension; and most importantly (c) highlight teens’ stress to enable them to generate emotional resonance and thus willingness to pursue the reading. Therefore in this paper, we propose to generate instructive questions from the multiple recommended articles to guide teens to read. Four solutions based on the neural encoder-decoder model are presented to tackle the task. For model training and testing, we construct a novel large-scale QA dataset named TeenQA, which is specific to adolescent stress. Due to the extensibility of question expressions, we incorporate three groups of automatic evaluation metrics as well as one group of human evaluation metrics to examine the quality of the generated questions. The experimental results show that the proposed Encoder-Decoder with Summary on Contexts with Feature-rich embeddings (ED-SoCF) solution can generate good questions for guiding reading, achieving comparable performance on some semantic similarity metrics with that of humans.

## 1. Introduction

### 1.1. Background

**Figure 1:**
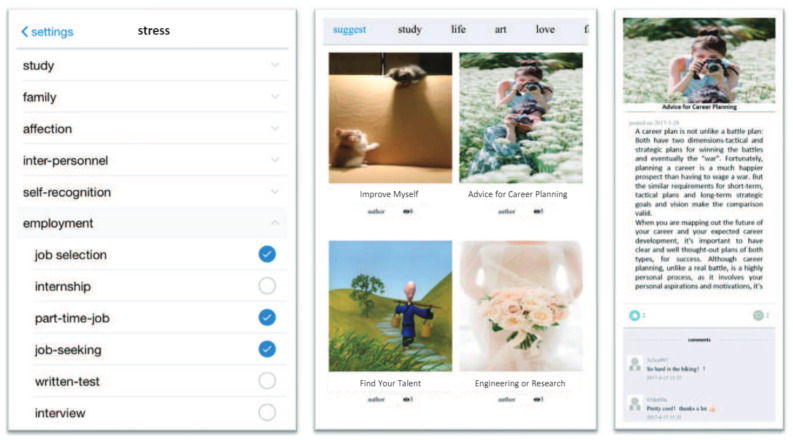
The interfaces of *TeenRead*. Teens first select several undergoing stress categories, then *TeenRead* recommends 4 articles based on the stress categories and teens’ instant stressful events.

With the rapid development of economy and society, teens are facing various psychological stress coming from study, family, love, peer relation, self-cognition and so on. Bibliotherapy has been proved to be an effective treatment method to deal with psychological stress [1,2,3]. It involves the reading of specific literature with the purpose of prevention, healing and rehabilitation. Through the feelings of resonance, intimation and/or apperception, readers could develop strength and courage to cope with their stress or mental problems [1,4]. As an adjunct therapy to psychological treatment, bibliotherapy is effective to people of all ages. However, the traditional bibliotherapy based on paper materials is ill-fitted in the present information era, especially for teens. On the one hand, bibliotherapy requires a lot of professional care, which is labor demanding and difficult to be carried out by teens themselves. On the other hand, teens who are deeply pressurized tend not to actively take the treatment for reasons of self-esteem. To address the limitations, the notion of e-bibliotherapy, which combines bibliotherapy with computers and internet, thus arose [5]. As a preliminary practice on E-bibliotherapy, Xin et al. have built an adolescent reading recommender system *TeenRead* which weekly recommends 4 articles to users based on the similarity between users’ stress categories and articles’ categories, as shown in Figure 1 [6]. The articles of *TeenRead* are from the psychology practitioners and some authorized users, who are willing to share literature as a means of mutual help and personal development, and the purpose of those articles is to address certain types of psychological stress of teens. Figure 1 gives a typical example of the article, which is shown in Table 1 in its entirety.

*TeenRead*’s automatic recommendation of appropriate articles to stressful teens alleviates the manual efforts on readings selection. This made one step towards online E-bibliotherapy. Beyond that, for the sake of better reading experience and more effective bibliotherapy, devising the instructive questions, instead of just devising the key phrases, from the multiple recommended articles to guide reading is also important and desirable. Such a question helps in three aspects:

(1) As psychological studies show, devising questions before reading can help attract readers’ attention [7].
(2) The instructive question conveys the essential message of the recommended articles so as to improve teens’ active comprehension. From this point of view, the question also serves as a headline.
(3) More importantly, considering the mission of *TeenRead* is assisting teens to ease stress by reading, the most effective way, which we believe can be achieved, is to react to and highlight teens’ stress. If the question (e.g., How to get along with parents who do not understand me?, what to do after quarreling with friends?) shows care and concern for stressful teens, they may feel resonance in emotion, and are thus willing to pursue the reading.

To this end, the study explores how to generate such instructive questions from the multiple articles recommended by *TeenRead*. For the four example articles in Figure 1, the generated instructive question could be *How can you plan your future career?* Formally, given *N* recommended articles $\mathrm{RA}=\{R_{1},R_{2},\cdots ,R_{N}\}$, our task is to generate an instructive question $T=\{t_{1},t_{2},\cdots ,t_{K}\}$ that covers the essential information of RA, where $t_{i}$ is an English word (1≤i≤K), $t_{K}=$‘?’ is the end mark of the question, and $t_{<n}=\left\{t_{1},t_{2},\cdots ,t_{n-1}\right\}$ represents all generated words before $t_{n}$. The task can be viewed as a seq2seq task, which can be addressed elegantly by a neural encoder-decoder model.

### 1.2. Challenges

In the literature, generating an instructive question is not explored to our knowledge, especially from multiple articles. This is a non-trivial task facing a number of challenges:

Task novelty and complexity. Although question generation (QG) task has been studied for years in the areas of reading comprehension [8,9,10,11] and question answering [12,13,14,15], most of them aim to generate questions either from the structured data (e.g., knowledge bases [16] and concept map [17]), or from a single sentence or passage [10,12,18,19,20,21,22], and the answer is short and objective. In this study, on the contrary, the answer is subjective, redundant and spreading over multiple articles, which requires more efforts to find the key information and join them to form the general question. This paper is the first trial to generate a question from multiple long articles, where every article is an independent answer to this question.

Question type diversity. Most of the previous work on QG focused on factoid questions [what, when, which, who, whom, where] [16], where an explicit mapping between questions and answers exists. In our task, more types of questions need to be considered, e.g., the causal and explanatory questions [how, why] asking for causal explanations, and methodological questions [what should, how can] seeking for advice and solutions, etc. For some types of questions, the connection between answers and questions is not that straightforward.

Information coverage. A question contains only a few words, but is required to cover the important information of multiple long articles. It demands effective synthesis and abstraction techniques, both of which remain a challenge for current NLP technology.

Statement fluency and diversity. The generated question should be readable with correct morphology and syntax. In addition, for the sake of attractiveness, the question expression should be various and flexible. We cannot imagine how bored users would be if all questions are in a fixed format of “*How can I*…?”. From this perspective, question generation based on heuristic rules and templates [8,20,23,24] is insufficient for our task.

### 1.3. Our Work

Inspired by the recent success of neural encoder-decoder models in handling sequence-to-sequence (seq2seq) tasks like machine translation [25,26], text summarization [27,28], speech recognition [29] and video captioning [30], we turn to encoder-decoder models for our instructive question generation task. The basic idea is that we first encode the multiple articles into a vector representation, then based on that the decoder generates the question word by word.

Construction of the effective encoder-decoder model heavily relies on the large-scale high-quality training dataset, whose examples are (question, articles) pairs. In the scenario of *TeenRead*, articles can be viewed as the independent answers towards the question under the specific stress category, because the majority of them are advice or solutions to teens’ stressful events (see Figure 1 and Table 1 as an example). Unfortunately, the currently available QA datasets [31,32,33,34,35,36] are not suitable for our task. On the one hand, the content of these datasets is not specific to adolescent bibliotherapy, hence their questions are unable to reflect teens’ stress. On the other hand, most of their answers are the brief factoid statements (see analysis in Section 3.2.5). Whereas in our task, we need a novel QA dataset, of which the question (serving as the instructive question) is specific to adolescent commonly encountered stress, and the answers are complex enough to help solving teens’ problems. Hence, before the design of the encoder-decoder model, we need to make efforts to build a large-scale suitable dataset.

Overall, the study makes the following three contributions:

(1) We propose a novel task generating instructive questions from multiple articles, whose aim is to guide teens to read in E-bibliotherapy. Four solutions based on the neural encoder-decoder model are presented to tackle the task: encoder-decoder with summary on outputs (ED-SoO), encoder-decoder with summary on inputs (ED-SoI) and encoder-decoder with summary on contexts with/out feature-rich embeddings (ED-SoC and ED-SoCF).
(2) We collected and constructed a novel large-scale QA dataset named *TeenQA* (Public at https://github.com/xinyx/TeenQA accessed on 10 October 2020) from Quora (https://www.quora.com/ accessed on 10 October 2020) for model training and testing. TeenQA contains 697,105 question–answer pairs covering seven categories of topics about teens’ commonly encountered problems and community-given solutions. TeenQA is naturally annotated, the topic of each question is accurately annotated by community users. In terms of question diversity, answer complexity and content subjectivity, TeenQA presents more challenges for question generation task (a detailed comparison between QA datasets is listed in Table 2).
(3) We conducted extensive experiments on 3 groups of automatic evaluation metrics and 1 group of human evaluation metrics. The automatic evaluation metrics evaluate the lexical similarity (BLEU, ROUGE, METEOR), human consensus (CIDEr) and semantic similarity (sentence similarity based on word embeddings) of the generated questions with that of original ones. While the human evaluation metrics evaluate whether the generated questions are well expressed and helpful for guiding reading or not. Our experimental results showed that ED-SoCF is able to generate fairly good reading guiding questions on human evaluation metrics and also performs best among the four solutions on automatic metrics: 27%, 18% and 13% higher than ED-SoO, ED-SoI and ED-SoC on lexical metrics, 48%, 53% and 38% higher on consensus metric, and its performance on some semantic metrics is comparable to that of humans (87.2 vs. 87.7).

The remainder of the paper is organized as follows. We review the related work in the areas of title generation and question generation in Section 2. The acquisition and analysis of our constructed dataset TeenQA is detailed in Section 3. Solutions for generating an instructive question from multiple articles, as well as their performance evaluation, are given in Section 4 and Section 5, respectively. Finally, we conclude the paper and point out more application scenarios of our solutions in Section 6.

## 2. Related Work

Our work is closely related to the existing studies on headline generation and question generation.

### 2.1. Headline Generation

One of the objectives of instructive questions is to convey the key information of reading articles, which is highly similar to a headline. Headline generation is a task of producing a condensed text summarization over one or multiple documents. From the perspectives of the granularity of the processing units, we divide the generation methods into three categories: extractive, non-neural abstractive and neural abstractive.

#### 2.1.1. Extractive Headline Generation Methods

In extractive methods, candidate sentences from original documents are extracted and put together to form the headline through sentence compression techniques. Four main lines of extractive headline generation techniques are developed and broadly employed.

(1) *Linguistic Rule-based Methods*

These methods make use of handcrafted linguistic rules for detecting and compressing important parts of documents. The representative example of this method is Hedge Trimmer [37], which built a parse and trim schema, and generated the headline for a news story by removing constituents from the parse tree of the lead (first) sentence of the news article until a certain length threshold is reached. In the work of Dorr et al., linguistically motivated techniques guide the choice of what constituents should be removed and retained [37].

The rule-based methods are simple and lightweight, and do not require prior training on a large scale corpus. However, as only limited candidate sentences (e.g., the first sentence [37]) are considered, rule-based approaches fail in capturing and exploring complex relations throughout the text for headline generation [38].

(2) *Statistics-based Methods*

As an improvement, these methods exploit statistic models for learning correlations between words in headlines and in documents, and work in a supervised learning setting with a large training corpus. A notable work is carried by Banko et al., which used the Naïve Bayes approach to learn the conditional probability of a word appearing in a headline given it appears in the document [39]: $P\left(w\in H|w\in D\right)=\frac{P(w\in H\wedge w\in D)}{P(w\in D)}$. To enforce the sentence structure and score candidate headlines, Banko et al. further computed the probability of word sequence through a bi-gram language model [39]. The overall probability of a candidate headline *H* consisting of word sequence ($w_{1},w_{2},\cdots ,w_{n}$) is computed as the product of the likelihood of (1) the terms selected for the headline, (2) the length of the resulting headline and (3) the most likely sequencing of the terms in the content set [39]:

$$P\left(w_{1},w_{2},\cdots ,w_{n}|D\right)=\prod_{i=1}^{n}P\left(w_{i}\in H|w_{i}\in D\right)\cdot P\left(len\left(H\right)=n\right)\cdot \prod_{i=2}^{n}P\left(w_{i}|w_{1},w_{2},\cdot ,w_{i-1}\right)$$

Similarly, Jin et al. selected headline words through the NBL, NBF, EM and TF-IDF methods, and then reordered them with a trigram language model [40]. Zajic et al. generated headlines for newspaper stories through a Hidden Markov Model [41]. The use of statistic models for learning pruning-rules for parse trees has also been studied by Knight et al. and Unno et al. [42,43].

Compared to rule-based methods, statistics-based methods rely on the availability of training corpus, and are more computationally expensive. However, due to the ability to learn from training data, statistics-based methods are robust and can be extended to different languages and domains, making it possible to generate cross-lingual headlines.

(3) *Summarization-based Methods*

In order to take advantage of text summarization techniques to do headline generation, these methods treat headlines as summaries with a very short length, and adapted traditional automatic text summarization techniques to address the headline generation task [38,44,45,46,47,48]. As the basic processing unit, salient sentences in the text are ranked for a summary based on certain features, such as term frequency [49], position in text [50,51], cue phrases [50,52,53], number of key words or title words in a sentence [51] and so on. Several machine learning algorithms like Naïve Bayes [54], decision trees [55] and semi-supervised learning algorithms [42,56] worked on the features to discover the most salient sentences. Then, single or multiple sentence compression techniques were applied to the salient sentences to generate a final headline. For instance, Zajic et al. built a system called Topiary that combines linguistically motivated sentence compression with statistically selected topic terms [57]. Colmenares et al. modeled headlines in a feature-rich space and took headline generation as a sequence prediction task using CRF model [58]. Filippova et al. compressed the sentence into a headline by deletion with LSTM [59]. Filippova built a word graph for multiple sentences and compressed them into a single sentence by finding the shortest paths [60].

The advantage of the summarization-based headline generation methods is that they treat text summarization and headline generation uniformly as the same task. Resorting to summarization techniques for headline generation may generate headlines of low quality when the compression ratio is lower than 10%. In addition, adopting summarization techniques is not applicable to cross-lingual headline generation.

#### 2.1.2. Non-Neural Abstractive Headline Generation Methods

The aforementioned extractive methods take sentences as the basic processing units, bringing great difficulty for information refining and rearrangement. With regard to this, non-neural abstractive methods select a set of salient phrases, concepts or events as the basic processing units according to specific principles during candidate extraction.

In these methods, the headline is generated word by word from scratch using sentence synthesis techniques and natural language generation techniques [61]. For instance, Tseng et al. mapped the category-specific terms of the news cluster into the common generic term based on the hypernym search algorithm with the help of WordNet, and took the generic term as headline [62]. Xu et al. extracted the keywords from the input document using novel word features derived from its relevant Wikipedia articles, then employed the keyword clustering based headline generation procedure to construct a document’s headline from the extracted keywords [63]. Genest et al. generated a guided summary using handcrafted abstraction schemes, which included rule-based information extraction, heuristic content selection and generation patterns [64]. Alfonseca et al. inferred the hidden event from the extracted syntactic patterns of news cluster based on a Noisy-OR Bayesian network, and then replaced the entity placeholders with the observed surface forms to generate the headline [65]. Sun et al. extracted the candidate events based on a bipartite graph of lexical chains and events, then obtained a headline by the graph-based multi-sentence compression model [66].

#### 2.1.3. Neural Abstractive Headline Generation Methods

Unfortunately, both of the above approaches have their own drawbacks. The extractive ones tailor human-written sentences to derive the final title, so they can generate more readable headlines than the abstractive ones. However, as sentences are usually sparse and longer than headlines, their generated headlines are usually less informative. Usually, headlines do not include the key words that are present in the source documents [38]. In contrast, the non-neural abstractive ones are based on phrases, concepts and events, which are much less sparse, so the generated headlines tend to be to-the-point. However, ensuring grammatical correctness and linguistic fluency of the generated headlines based on a set of phrases and concepts is challenging [66].

To overcome the limitation of extractive and non-neural abstractive methods, the neural-based headline generation methods recently attracted a lot of attention due to the success of neural sequence-to-sequence (seq2seq) model on machine translation [25,26,67,68,69], text summarization [27,28,70,71,72], speech recognition [29] and video captioning [30]. The processing unit of the model is at the document-level. It first encodes the input text into a context vector representation. The context representation in turn constrains the output of the target sequence.

Kalchbrenner et al. first applied this model to machine translation, in which the input sentences are mapped into vectors using convolutional neural networks so the sequence information is lost [73]. Later on, Sutskever et al. substituted the encoder and the decoder with both LSTM and implemented the first pure neural translation system that outperformed the phrase-based statistic machine translation by a sizeable margin [25]. A potential issue with this encode-decode model is that when compressing the long sequence into a fixed-length vector, the key concepts will be severely lost. To address this issue, Bahdanau et al. proposed the attention mechanism which allows the decoder to automatically search for the relevant parts of the source sequence as the context [26].

Many researches have been done to explore different attention mechanisms. Luong et al. examined two classes of attention mechanisms: a global approach that considers all hidden states of the encoder when deriving the context vector, and a local one that chooses to focus only on a small subset of the source positions at a time [67]. The experiment showed that the global attention with dot alignment and the local attention with predictive alignment works best. Lopyrev adopted the former best attention mechanism but implemented it in two different ways: the complex attention which remains unchanged, and the simple attention that split the hidden states into 2 sets to separately compute the attention weight and decode [27].

In the headline generation task, Rush et al. firstly employed an encoder-decoder model to generate a headline from the lead (first) sentences of news articles [70]. Its encoder is an attention-based convolutional neural network, and the decoder is a feed-forward neural network language model. The model was trained on a large amount of news headlines and selected recapitulative sentences. Following the strategy, Lopyrev generated news headlines with both RNN for encoder and decoder [27]. To capture the syntactic properties of the sentence, Tai et al. proposed a Tree-LSTM that generalizes LSTMs to tree-structured network topologies [74]. Based on Tree-LSTM, Takase et al. incorporated the structural syntactic and semantic information into encoders [75]. Chopra et al. employed RNN as the encoder, and incorporated the position information of words, which showed significant improvement [71]. Furthermore, many advanced features and mechanisms were proposed to enhance the performance of the encoder-decoder model for text summarization and headline generation. Nallapati et al. restricted the decode-vocabulary of each mini-batch to the words in the source documents of that batch and the most frequent words in the target vocabulary to reduce the soft-max penalty [28]. It also captured the keywords using a feature rich encoder, used switching generator-pointer to handle the out-of-vocabulary (OOV) problem and applied a word-level encoder and a sentence-level encoder to capture the hierarchical document structure. Gu et al. proposed a novel COPYNET model to explore the copying mechanism, which located a certain segment of the input sentence and puts the segment into the output sequence [72].

Even with LSTM and the attention mechanism, it is still hard for the encoder-decoder model to capture the document/paragraph-level information. To move towards this task, Li et al. proposed a hierarchical neural auto-encoder to preserve and reconstruct multi-sentence paragraphs [76]. They used an LSTM model to encode a paragraph into an embedding for sentences and words composing it, then decoded this embedding to reconstruct the original paragraph. Tan et al. attempted to generate news headline by encoding different summaries of a news into a summary representation and generating the output with a hierarchical attention mechanism [61].

### 2.2. Question Generation

Question generation aims to generate questions from a given sentence or paragraph. One key application of question generation is in the area of education for reading comprehension [8,9,10]. Combining question generation and question answering as dual tasks also enables to improve question answering systems in natural language processing fields [12,13].

Three typical methods have been developed on question generation task, which are rule-based, template-based and neural [19,77,78].

#### 2.2.1. Rule-Based Question Generation Methods

Rule-based approaches rely on well-designed rules for declarative-to-interrogative sentence transformation based on deep linguistic knowledge [79,80]. Heilman et al. used manually written rules to generate multiple questions from a sentence, and then ranked the questions through a logistic regression model trained on a tailored dataset consisting of labeled outputs [9].

#### 2.2.2. Template-Based Question Generation Methods

In addition to rule-based approaches which exploit syntactic roles of words in generating questions, another group of research turns to manually construct question templates and then applies them to generate questions [23,81,82]. Lindberg et al. introduced a template-based approach which incorporated semantic role labels to generate natural language questions to guide online learning [23]. Labutov et al. used crowdsourcing to collect a set of templates for the text and then ranked the relevant templates for the text [20]. It encoded the original text in a low-dimensional ontology, and then aligned the question templates to that space to get the top relevant templates. Chali et al. generated questions from a topic, associated with a body of texts containing topic-related useful information. Then, questions are generated by exploiting the named entity information and the predicate argument structures of the sentences present in the body of texts [19]. Serban et al. generated simple factoid questions from logic triple (subject, relation, object), where structured representation is mapped to natural language text [16].

Both rule-based and template-based question generation methods rely on manually generated rules and templates, and the generated questions are constrained by the human-designed transformation which makes it hard to scale to other domains.

#### 2.2.3. Neural Question Generation Methods

To overcome the limitations of the above methods, currently many researches have shifted to generating questions with the encoder-decoder model. Serban et al. [16] proposed a neural network model to generate the factoid questions from FreeBase KB [83]. Instead of generating from structured triples, Zhou et al. generated meaningful and diverse questions from natural language sentences, where the encoder is enriched with answer position and lexical features [21]. Yuan et al. explored the training skill using a combination of supervised and reinforcement learning [22]. Without regards to the answer information, Du et al. investigated the effect of encoding sentence- vs. paragraph-level information, of which the sentence-level mode achieved the state-of-the-art performance [10].

Neural question generation methods use deep sequence-to-sequence learning approach to generate questions. They are fully data-driven and provide an end-to-end solution without the guidance of rules or templates [10].

### 2.3. The Recently Released QA Datasets

Table 2 lists some recently released popular QA datasets for question generation, question answering and reading comprehension, where the majority of the questions belong to factoid questions.

(1) *SQuAD* [31] collects 100 K question–answer pairs from crowdworkers on 536 Wikipedia articles. The answer to each question is a segment of text from the corresponding passage.
(2) *MS MARCO* [32] contains 100 K questions, 1 M passages and links to over 200 K documents. The questions are real queries issued through Bing. The passages are extracted from the web documents returned by Bing, and the answers are human generated based on the related passages.
(3) *TriviaQA* [33] includes 95 K question–answer pairs from 14 trivia and quiz-league websites, and collects the textual evidence documents from Bing search results and Wikipedia articles. There are on average 6 evidence documents for deriving the answer to a question.
(4) *WikiQA* [35] contains 3 K questions sampled from Bing. Each question is associated with a Wikipedia page based on the user clicks. All sentences of the page summary are extracted as candidates and are labeled on whether the sentence is the correct answer of the questions by crowdsourcing workers. Overall, there are 29 K sentences obtained.
(5) *MCtext* [84] has 660 fictional stories created by crowdworkers and 4 multiple-choice questions per story.
(6) *NewsQA* [34] collects 100 K question–answer pairs from crowdworkers on 10 K news articles from CNN, where the answer is also a span of text from the corresponding articles.
(7) *rc-data* [85] includes 287 K newspaper articles from CNN/Daily Mail news. Based on this, 1 M cloze questions are constructed by replacing the entities with placeholders.
(8) *SimpleQuestions* [36] consist of 108 K questions written by human English-speaking annotators based on the corresponding facts in Freebase, where a fact is of the form (*subject-relationship-object*), and the answer is *object*.
(9) *TeenQA* is the constructed QA dataset in this paper. It contains 697,105 questions and 1,962,895 answers (2.8 answers per question on average) specific for teens’ stress which are generated by the community users of Quora. In addition to questions and answers, TeenQA includes other tag information: the topic and *follows* of the question, the *upvotes*, answerer and answer date of the answer and the description of the answerer.

The QA pairs in TeenQA are strikingly different from the existing QA datasets in the aspect of question diversity, answer complexity and content subjectivity, which presents a great challenge for question generation task.

### 2.4. The Novelty of Our Work

The model proposed in this paper is inspired by the success of neural question/headline generation in previous work. This is the first trial to migrate question auto-generation to the field of bibliotherapy, which requires the generated questions not only to be well expressed but also to be able to attract teens’ attentions, convey the essential message of multiple articles and highlight teens’ stress to raise emotional resonance. Beyond that, our model investigates the possibility of capturing the key information from multiple independent articles in a neural-abstractive way, which is barely studied in the literature. To train and test the model, we also construct a novel large-scale topic-specific QA dataset TeenQA.

## 3. Dataset and Analysis

We collected and constructed a large-scale topic-specific naturally annotated QAs dataset, focusing on Teens’ commonly-encountered problems. In this section, we first introduce the collection procedure, then analyze the dataset in detail to show its applicability to our task. Finally, we explore the possibilities to solve other tasks with TeenQA.

### 3.1. Data Collection

The question–answer (QA) pairs of TeenQA are crawled from Quora (www.quora.com accessed on 10 October 2020). Quora is a popular question-and-answers website created in 2009, where questions are asked, followed and answered by community users. We choose Quora as a data source for the following 2 reasons:

Topic-specific questions. Although knowledge in Quora is open-domain, each asked question is categorized by community users into several topics. Given teens’ mostly concerned topics, we could collect the relevant QA pairs.

High Quality. We can extract the QA pairs of high quality through question’s *follows* tag and answer’s *upvotes* tag. In general, more *follows* mean the question is paid more attention to. Similarly, more *upvotes* indicate the answer is accepted with higher quality.

#### 3.1.1. Question Topic Seeds

**Figure 2:**
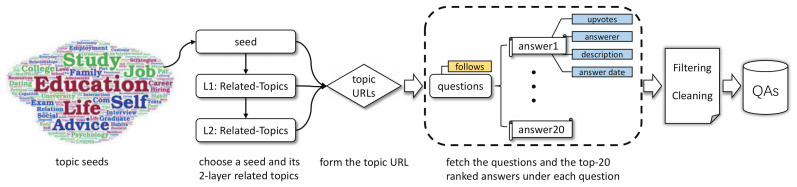
The crawling process of TeenQA. Quora provides a related-topic-list for each topic, we recursively crawl 2-layer related topics of the topic seed.

According to teens’ commonly encountered stress categories, we manually select 66 question topics existing in Quora as seeds, each seed corresponding to a stress sub-category (seen in Table 3, one stress sub-category may correspond to several seeds). To enrich the dataset, we also crawl the QA pairs belonging to the related topics of the seeds with the help of Quora’s topic recommender system. Figure 2 illustrates the crawling process.

#### 3.1.2. Data Filtering and Cleaning

While crawling, we filter and clean the data in 3 aspects.

Question Length. Short questions tend to be more general, consequently to be harder to derive from answers. On the other hand, longer questions are more specific, but it is not suitable as an instructive question and may not have enough answers. To balance between generalization and specificity, we limit the question length to be 4 to 15 words.

Upvotes Number. For each question, Quora ranks the answers based on answers’ *upvotes* and freshness. It is extremely difficult to crawl down all answers to a question restricted by Quora’s display strategy, so we only obtain its first 20 answers in the answer panel, then from which only up to 4 answers with the most *upvotes* numbers are preserved to make sure the quality of answers. We choose to select 4 answers because there are exactly 4 articles for each instructive question in *TeenRead* considering users’ reading habits and the UI design.

Answer Body. As an informal community QA site, Quora does not constrain users’ writing styles. As a result, the answers are colloquial, containing plenty of unstructured contents, irregular symbols and white spaces. To normalize the data, we only keep the plain text, map the punctuation marks into English styles, remove the meaningless split lines and add ‘.’ to sentences if the end punctuation is missing.

In this way, we obtain a QA dataset *TeenQA*, containing 697,105 questions and 1,962,895 answers (2.8 answers per question on average). Table 4 presents an example of QA pairs in TeenQA, in which the answers are sorted by *upvotes* numbers in descending order.

### 3.2. Data Analysis

#### 3.2.1. Feasibility of TeenQA

On the one hand, the answers of TeenQA can be reasonably regarded as the articles of *TeenRead*, since in the scenario of *TeenRead* the majority of the articles are the independent solutions or advices to teens’ problems.

On the other hand, the questions of TeenQA can also serve as instructive questions for the articles. As discussed in Section 1.1, an instructive question to guide teens to read should meet 3 requirements: attracting teens’ attention, conveying the essential message of the readings and highlighting teens’ stress. For TeenQA, the first two requirements are satisfied, since the question headline can naturally attract attention of teens who are in need, and the question is also the refined summarization of answers. To validate if the question can highlight teens’ stress, we randomly sampled 1000 examples (it is also used as a testing dataset in our experiments) to ask 3 psychology-related researchers to label whether the question is related to teen’s stress or not, and which category of stress in Table 3 could the question reveal. The result showed more than 95% of the questions are correlated with teens’ stress, and the Macro-F1/Micro-F1 score for classification is 80.7%/91.3%.

Above all, it is reasonable to assimilate the answers and questions of TeenQA to the articles and instructive questions of *TeenRead* in our task.

#### 3.2.2. Distribution of Stress Categories

Table 5 shows the distribution of stress categories in TeenQA. *study* and *employment* are the most significant stress categories, which together take up more than a half of all QA pairs. The other 5 stress categories are almost equally sized. This distribution is consistent with the stress situation of adolescents.

#### 3.2.3. Lengths of Questions and Answers

**Figure 3:**
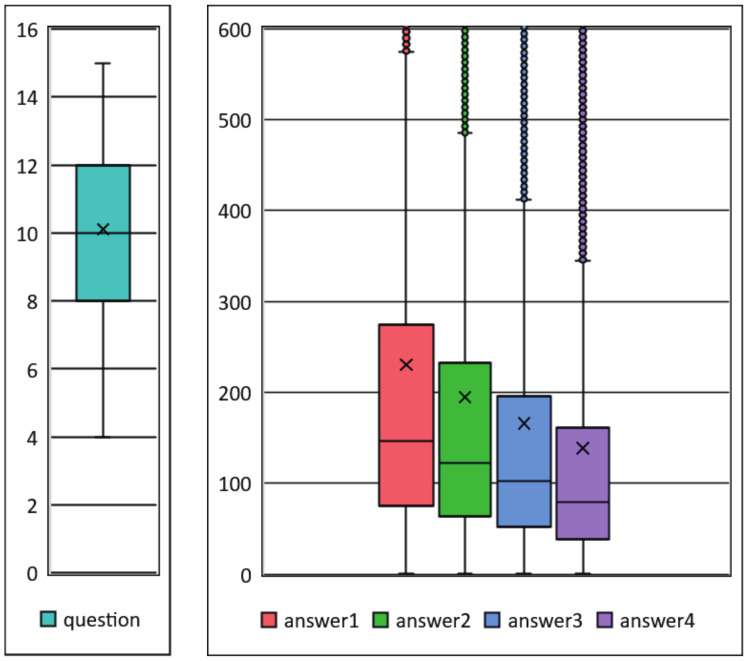
Lengths of QAs in TeenQA.

As shown in Figure 3, the average length of questions in TeenQA is around 10 words, which is suitable for serving as an instructive question. Answers receiving more *upvotes* usually contain more words, the average lengths of four answers are 195, 160, 146 and 138, respectively. Considering all the 4 answers, their average length is around 165 words. In the boxplot, the lines from the bottom to the top denote the minimum, the first quartile, the median, the third quartile and the maximum of the length. × mark denotes the average value. Dots above the top line denote outliers, which could be very long.

#### 3.2.4. Number of Questions’ Follows, Questions’ Answers and Answers’ Upvotes

**Figure 4:**
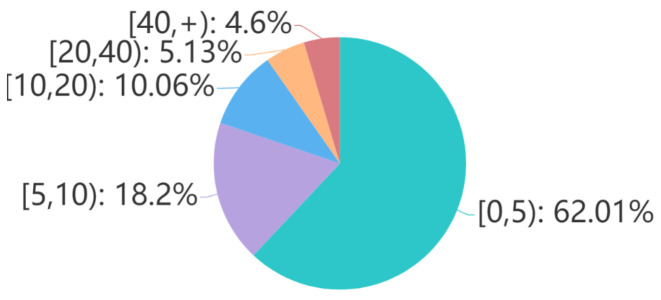
Number of questions’ *follows*.

**Figure 5:**
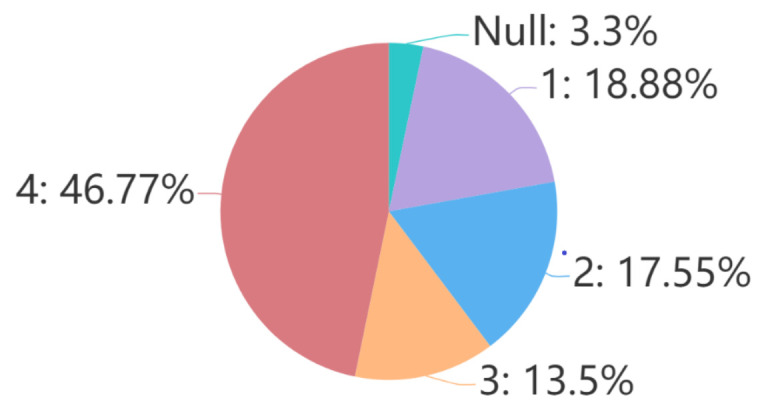
Number of answers.

Figure 4 and Figure 5 and Table 6 list the numbers of questions’ *follows*, questions’ answers and answers’ *upvotes*, respectively.

Question’s *follows* number can be used to measure users’ attention degree to the question and the corresponding topic. In order to know more about teens’ stressor events, E-bibliotherapy and education practitioners should pay more attention to those questions with large *follows*, especially those with more than 40 follows, which accounts for 4.6% of all.

Similar to question’s *follows* number, answer’s *upvotes* number reflects the acceptability and the quality of the answer. Table 6 shows the relation between answers’ ranks and answers’ *upvotes*, through which we can see that there are 1,962,895 answers in total, and 6.2% of them have more than 40 *upvotes*.

#### 3.2.5. Types of Questions

Diversity of question types is an important criterion of dataset’s feasibility. As the training dataset is for E-bibliotherapy, TeenQA should accommodate different kinds of questions to help generating appropriate guiding questions. We extend the categorization of question types made for question answering systems [86] to consider the following six types of question for E-bibliotherapy:

Factoid questions [what, when, which, who, whom, where]. (The words in [ ] are the typical question words of each type of questions.) These questions are simple and fact-based, their answers are a short span of words, entity or sentence.

*List questions*. This type of question can be decomposed into several factoid questions, e.g., *what are the most popular programming languages?*

*Hypothetical questions [what if, how if]*. Hypothetical questions ask for answers based on a hypothesis, which are hard to answer because they are highly subjective to questions and the answers are not specific.

*Confirmation questions [is, will]*. This type of question requires answers in the form of *yes* or *no*. In Quora, answerers usually provide detailed explanations on why they choose *yes* or *no*.

*Causal and Explanatory questions [how, why]*. This type of question asks for answers explaining one phenomenon.In order to explain it clearly, the answers tend to be quite long.

*Methodological questions [what should, how can]*. This type of questions are highly valuable in our task. When teens encounter problems, they tend to pose questions for detailed solutions or advices. However, this type of question is the hardest question to derive from multiple answers. It is because the answers are completely subjective to answerers, and the key information of the question is sparsely spreading across different answers.

Based on the above classification, we analyze the distribution of question types in TeenQA in Table 7 using the randomly sampled 1000 examples in Section 3.2.1. As we wish, all types of questions are present with considerable proportions and the methodological questions take up the most in TeenQA, accounting for 27.1%.

As a comparison, we also analyzed the question types of datasets SQuAD, WikiQA and MARCO, which are also used in question generation tasks [10,13]. We randomly sampled 100 questions from them respectively and classified them into the 6 categories listed above. The results show that the types of questions in the three datasets are mainly concentrated in the factoid category, with a ratio of 82%, 83% and 79%, respectively. The proportion of causal questions are only 5%, 9% and 4%, respectively. As for methodology questions, for the most valuable questions for our task, the proportion is all smaller than 3%. Comparing with Table 7, we can find that the question type distribution of TeenQA is more diverse.

### 3.3. More Applicable Scenarios of TeenQA

In addition to the instructive question generation task, TeenQA could also be applied to other scenarios:

1. General Question Generation for Reading Comprehension.The existing QA datasets for reading comprehension (RC) in Table 2 are either for answering the question based on context(s) or for generating questions from sentences or short paragraph(s). However, generating an abstractive question covering the main idea of the whole long text is a more comprehensive measurement for RC, which is far more difficult in that users should understand all textual information first. Furthermore, generating a high-level general question from multiple documents around the same topic is even more tough. To this end, TeenQA is the first large scale dataset supporting question generation from both single document and multiple documents. TeenQA provides sufficient (general question, answer(s)) pairs, and these answers can be seen as stand-alone documents to derive the general questions.
2. Adolescent Stress Analysis. TeenQA contains nearly 700 K topic-specific questions, related to the typical stress categories of adolescents about *study, family, peer relation, romantic relation, self-cognition, employment* and *life*. The attention degree of each question can be inferred from question’s *follows* number. This makes it possible for the quantitative study of adolescent stress. We hope that TeenQA could help psychologists and education practitioners better understand and solve teens’ stress problems.

## 4. Proposed Models

### 4.1. Problem Formulation

Given *N* recommended articles $\mathrm{RA}=\{R_{1},R_{2},\cdots ,R_{N}\}$, our task is to generate an instructive question $T=\{t_{1},t_{2},\cdots ,t_{K}\}$ that covers the essential information of RA, where $t_{i}$ is an English word (1≤i≤K), $t_{K}=$‘?’ is the end mark of the question, and $t_{<n}=\left\{t_{1},t_{2},\cdots ,t_{n-1}\right\}$ represents all generated words before $t_{n}$. We can describe the task in a probabilistic framework:

(1) $$\hat{T}=\arg\max_{T}P\left(T|\mathrm{RA}\right)$$

(2) $$P\left(T|\mathrm{RA}\right)=\prod_{n=1}^{K}P\left(t_{n}|t_{<n},\mathrm{RA}\right)$$

The task can be viewed as a seq2seq task, which can be addressed elegantly by a neural encoder-decoder model [25,26].

### 4.2. Overview of the Encoder-Decoder Model

For an input sequence $X=\{x_{1},x_{2},\cdots ,x_{m}\}$, where $x_{i}$ is the one-hot presentation of words from vocabulary V, the encoder-decoder model first encodes X into a context vector representation c, then decode c to generate the output sequence $Y=\{y_{1},y_{2},\cdots ,y_{n}\}$ word by word based on model parameters θ and the precedently generated tokens $y_{<i}$: $P\left(Y|X;\theta \right)=\prod_{i=1}^{n}P\left(y_{i}|y_{<i},c;\theta \right)$. Y with the highest conditional probability is chosen as the final output: $\hat{Y}=\arg\max_{Y}P\left(Y|X;\theta \right)$.

Encoder. The encoder encodes the input sequence into a context vector representation c. There are many variants of encoders, of which Recurrent Neural Network (RNN) [87] with LSTM [88] units is one of the mostly used one owing to its capacity for dealing with long sequence. Before encoding, the input words X are mapped to low-dimensional real-valued embeddings E, which carries the semantic information of words: $E=\left\{e_{1},e_{2},\ldots ,e_{m}\right\}=W_{emb}\cdot X$, where $W_{emb}\in R^{Dim\times |V|}$ is the embedding matrix, Dim is the embedding dimension and |V| is the vocabulary size.

Then RNN encoder calculates the hidden state $h_{t}$ of each word based on the word embedding $e_{t}$ and the former hidden state $h_{t-1}$: $h_{t}=f\left(e_{t},h_{t-1}\right)$, where *f* indicates the function of RNN unit.

After all hidden states are obtained, the context vector representation is calculated as: $c=g(\left\{h_{1},h_{2},\ldots ,h_{m}\right\})$, where *g* is the context function. Usually, we make $g=h_{m}$ for the reason that the last hidden unit can be seen as the compression of X.

Decoder. To better capture the sequence information, we also use LSTM-RNN as the decoder. It generates the output based on the hidden state $s_{t}$ and the context vector c: $y_{t}=\arg\max_{y}P\left(y|s_{t},c;\theta \right)$, where $s_{t}=\psi \left(s_{t-1},y_{t-1}\right)$. ψ is the function calculating the current hidden state with respect to the last hidden state and the last output.

Attention. The use of fixed-length context vector makes it hard to cope with long input sequences. Bahdanau et al. proposed the attention mechanism which allows the decoder to automatically search for relevant parts of the source sequence as context [26]. Consequently, the calculation of the current output is changed to:

(3) $$\begin{array}{c} y_{t}=\arg\max_{y}P\left(y|s_{t},c_{t};\theta \right),\;\;\;\;\mathrm{where}\;\;\;\;c_{t}=\sum_{i=1}^{m}\alpha_{i}^{t}h_{i} \end{array}$$

$\alpha_{i}^{t}$ is the attention weight on the *i*th input word while decoding $y_{t}$.

Training. The encoder-decoder model is mostly trained with minimum negative log likelihood (NLL) strategy [89]:

(4) $$L_{NLL}\left(\theta \right)=\sum_{(X,Y)\in D}logP\left(Y|X;\theta \right)$$

where D is the training dataset.

Limitations in instructive question generation. Although the encoder-decoder model has demonstrated great success in handling sequence-to-sequence tasks like machine translation, text summarization, speech recognition and video captioning, it has limitations in instructive question generation from multiple articles.

Firstly, even with the attention mechanism, the encoder-decoder model is still insufficient in coping with long texts, because too many textual contents makes the model hard to train and hurts the performance [61,76,90].

Secondly, the content fed into the encoder should be self-consistent so that the context vector makes sense. However, in our task, the multiple articles are given by different users. Even though they are targeted at the same question, they could also differ a lot from each other, and in some cases even contain diametrically opposed subjective contents and opinions. How to encode these diverse contents into a context representation vector and extract their general idea in the meanwhile are problems worthy of deep investigation.

We introduce our 4 solutions to address the long-text and diverse-content issues in the following subsections.

### 4.3. Solutions

#### 4.3.1. Solution 1: Encoder-Decoder with Summary on Outputs (ED-SoO)

**Figure 6:**
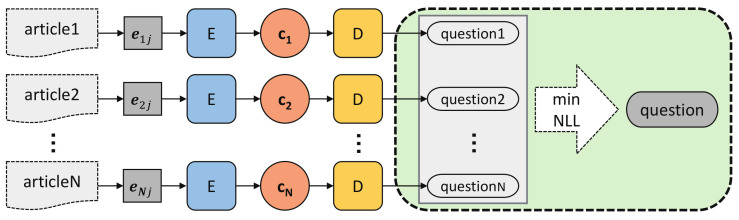
Solution 1: ED-SoO. E, D denote the encoder and decoder, $e_{ij}$ is the word embedding of the *j*th word in the *i*th article, $c_{i}$ is the context vector of the *i*th article.

Confronted with multiple articles, our first solution is to generate an instructive question from each individual article, and then pick up the one with the minimum negative log likelihood (NLL, Equation (4)) loss as the final question (Figure 6). Our basic encoder-decoder model *ED* implements the encoder and decoder both with a 3-layer LSTM, each layer having 512 hidden units. Like Lopyrev [27], a simple yet efficient attention mechanism splits hidden units into 2 parts: the first 472 units are for computing the context and decoding words, and the last 40 units are for computing the attention weight. In the decoding phrase of training, we use teacher forcing strategy [27] which randomly replaces the word of the training targets with the generated word at last timestep with 10% probability.

**Figure 7:**
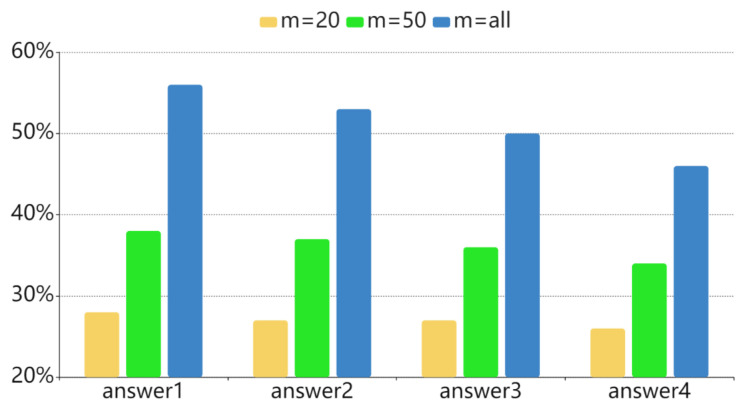
Words overlap between questions and the first *m* words of answers in TeenQA.

To handle the long text, we perform lead summarization to extract the first *m* words of each article as input based on our observation that most of the useful information tends to appear in the first small part of the articles (Figure 7).

Solution 1 addresses the diverse-content issue at the question-level rather than article-level. It has two problems:

*Low information density*. A single article may not have enough information to derive the question. Take TeenQA for example, the average word overlap between a question and an article (answer) is around 50% (Figure 7).

*Content incompleteness*. The single article addresses user’s problem from a one-sided perspective, affecting the model to generate a biased question. Only synthesizing all articles could we capture the essential point of the problem to generate a more precise question.

#### 4.3.2. Solution 2: Encoder-Decoder with Summary on Inputs (ED-SoI)

**Figure 8:**
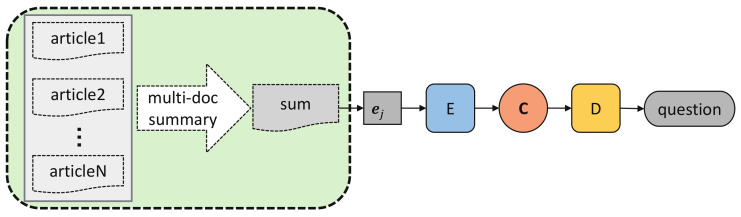
Solution 2: ED-SoI. E, D and C denote the encoder, decoder and context vector, respectively. $e_{j}$ is the word embedding of the *j*th word in the multi-document summary.

To overcome the problems of Solution 1, our second solution (Figure 8) is to first synthesize multiple articles with the help of multi-document summarization techniques, and then encode the summary into an overall context vector, based on which the decoder obtains an output as the question for these articles.

Multi-document summarization could identify and eliminate the redundancy across articles, recognizes novel information in different articles and try to make the final summary both coherent and complete. It could be very applicable to deal with the low information density and content incompleteness problems of Solution 1. In the study, we apply 6 different multi-document summarization techniques to summarize the *N* input articles into an *s*-word text.

(1) *Coverage summarization*. This is the baseline summarization method. It successively takes the lead sentences from all articles until the summary length threshold is reached.
(2) *Centroid summarization* [91]. Centroid-based method first constructs a centroid of the articles, which consists of words whose TF-IDF scores are above a pre-defined threshold. The salience of each sentence is computed as the weighted average of the similarity between the sentence and the centroid, sentence position within an article and the similarity between the sentence and the first sentence of the article.
(3) *TextRank summarization* [92]. TextRank is a graph-based sentence ranking model where each sentence is added as a vertex and the sentence similarity (e.g., words overlap) is added as an edge between sentences. After the graph-based ranking algorithm converges, we can sort sentences based on their final scores.
(4) *ILP summarization* [93]. Integer Linear Programming (ILP) method takes document summarization tasks as a combinatorial optimization problem, whose optimization goal is to cover as many word n-gram concepts as possible within the length constraint.
(5) *ClusterCMRW summarization* [94]. Cluster-based Conditional Markov Random Walk (ClusterCMRW) method first detects the theme clusters in articles, and then incorporates the cluster-level information and the sentence-to-cluster relationship to compute the saliency score of the sentences based on a Conditional Markov Random Walk model.
(6) *Submodular summarization* [95]. Submodular method formalizes the document summarization task as submodular function maximization problem under the budget constraint, where the information coverage, non-redundancy and diversity are reflected in the classes of submodular functions.

**Figure 9:**
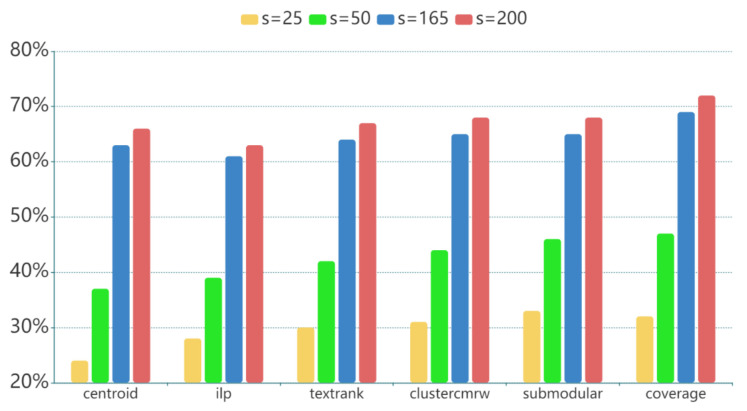
Words overlap between questions and the first *s* words of the multi-doc summary of 4 answers in TeenQA.

Comparing Figure 9 with Figure 7, multi-document summarization of 4 articles obtains a significant gain of information density. The content incompleteness could also be solved to a large degree by combining all articles. There remains a serious problem in multi-document summarization that it assumes the articles are coherent with each other so as to make sure the summary is meaningful. However, this assumption does not always hold when the articles are subjective with quite contradictory contents. These non-coherent contents in the same summary will inevitably confuse the model. To avoid this discordance, we further apply an encoder-decoder model with summary on contexts to pick out the general coherent information among all the articles.

#### 4.3.3. Solution 3: Encoder-Decoder with Summary on Contexts (ED-SoC)

**Figure 10:**
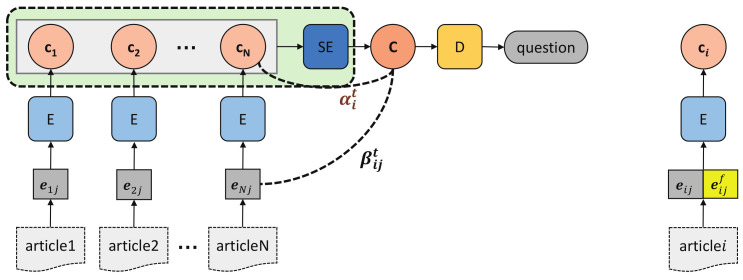
The ED-SoC solution and ED-SoCF solution. SE denotes the summary encoder that sequentially encodes $c_{1}$, …, $c_{N}$ into an overall context vector C. $e_{ij}$ and $e_{ij}^{f}$ are the word embedding and feature embedding of the *j*th word in the *i*th article. $\alpha_{i}^{t}$ and $\beta_{ij}^{t}$ is the article-level attention weight and the word-level attention weight.

Solution 3 independently encodes each article into context vector representation $c_{i}$ to preserve its content as much as possible, and then summarizes them through a summary encoder *SE* and a hierarchical attentional decoder (the left in Figure 10).

To get rid of the influence of the subjective contents, we take the first *m* words of each article as input (the same as ED-SoO), through which we can also obtain a higher words overlap between question and input sequences (it reaches 71% in TeenQA when m=50). In the encoding phrase, we use the same encoder to encode each article into a vector representation $c_{i}$. While decoding, the summary encoder *SE* (which is a single-layer LSTM) summarizes $c_{1}$, …, $c_{N}$ into a summary representation C, which can be regarded as the context representation of all articles. Then a decoder is used to generate output words based on C and the hierarchical attention mechanism that picks out the appropriate parts across all articles as decoding context. The decoding context vector $C_{t}$ for generating $y_{t}$ in Equation (3) can be recalculated as:

(5) $$\begin{array}{cc} C_{t} & =\sum_{i=0}^{N}\sum_{j=1}^{m}\alpha_{i}^{t}\beta_{ij}^{t}h_{ij} \end{array}$$

(6) $$\begin{array}{cc} \alpha_{i}^{t} & =\frac{exp(a\left(s_{t},c_{i}\right))}{\Sigma_{k=1}^{N}exp\left(a\left(s_{t},c_{k}\right)\right)} \end{array}$$

(7) $$\begin{array}{cc} \beta_{ij}^{t} & =\frac{exp(a\left(s_{t},h_{ij}\right))}{\Sigma_{k=1}^{m}exp\left(a\left(s_{t},h_{ik}\right)\right)} \end{array}$$

where *N* is the article number, *m* is the length of each article, $h_{ij}$ is the hidden state of the *j*th word in the *i*th article, $\alpha_{i}^{t}$ is the article-level attention weight indicating how much attention should be paid on the *i*th article, $\beta_{ij}^{t}$ is the word-level attention weight indicating how much attention should be paid on the *j*th word of the *i*th article.

#### 4.3.4. Solution 4: ED-SoC with Feature-Rich Embeddings (ED-SoCF)

We enhance Solution 3 by embedding a set of features, such as topic feature, articles’ upvotes feature, TD-IDF feature, as well as two linguistic features (POS and NER). All the features are concatenated with the embedding of input words.

(1) *Topic*. In the task, the generated question should be highly relevant with users’ stress, which could be revealed by the question topic. As shown in Figure 2, each question in TeenQA is assigned with a topic. We tokenize the topic into tokens, remove the stop words, look up their word embeddings and take their average embedding as the topic feature of each article word.
(2) *Upvotes*. Empirically, upvotes number reflects article’s quality, and the model should learn to pay more attention to the words from articles of higher quality. Since the answers in TeenQA are sorted by upvotes, we simply denote the upvotes feature with a 4 dimension one-hot vector that indicates which answer the word is from.
(3) *TF-IDF*. TF-IDF is a numerical statistic that reflect how important a word is to a document in a collection or corpus. TeenQA is crawled from Quora, adding the TF-IDF feature to each word embedding could highlight the key word and help generating Quora-like questions. To do this, we first calculate the TF-IDF value of each word, then divide the value into 5 buckets with equal size, denoted with a 5 dimension one-hot embedding.
(4) *POS*. Parts of Speech (POS) indicates the lexical category that a word belongs to. The POS sequence brings in grammatical information for input sentences, with the help of which we can improve the syntax correctness of the generated questions. To obtain the POS embedding of the word, we first assign each word with its POS tag using NLTK toolkit (http://www.nltk.org/ (accessed on 10 October 2020)) and then train a word2vec model implemented in gensim (https://radimrehurek.com/gensim/models/word2vec.html (accessed on 10 October 2020)) to cast POS tags into 20-dimension embeddings.
(5) *NER*. Named Entity Recognition (NER) locates the named entities into several pre-defined categories, which is of great help for determining the correct question words. We use NLTK toolkit to find six types of named entities: PERSON, ORGANIZATION, LOCATION, GPE, FACILITY, GSP, and train them into 10-dimension embeddings using a word2vec model as well.

Finally, we concatenate the word embedding with the features embedding (the right in Figure 10): $\tilde{e_{ij}}=\left[e_{ij},e_{ij}^{f}\right]$, where $e_{ij}$ is the original embedding of the *j*th word in the *i*th article, and $e_{ij}^{f}$ is the best combination of Topic, upvotes, TF-IDF, POS and NER embeddings.

## 5. Experiments

We conduct four experiments to examine the performance of our instructive question generation solutions. The experimental setup (including dataset, implementation details and performance metrics) and experimental results are reported in this section.

### 5.1. Experimental Set-Up

#### 5.1.1. Dataset

We use the questions and answers in TeenQA to train and test the model instead of the instructive questions and articles in TeenRead. In order to be on par with TeenRead and to eliminate the influence of different number of answer, we only take samples that have 4 answers in TeenQA as the experimental dataset D. As Figure 5 shows, D accounts for 46.77% of the total, i.e., 326,053 (question, 4 answers) pairs.

In the data cleanup phase, we (1) delete the leading digits or symbols of each list item in answers. For each obtained single/multi-document summary, we (2) lowercase the summary and the question, (3) remove split lines, (4) tokenize the text with NLTK, (5) delete all symbols except the single quotation mark and (6) replace digits with ‘#’. Finally, we randomly split D into 3 parts: 324,053 pairs for training, 1000 pairs for validating and 1000 pairs for testing.

#### 5.1.2. Implementation Details

*Word Embedding Initialization*. We keep the 40,000 most frequent words as vocabulary V and replace the others with 〈unk〉 symbols. The token 〈eos〉 indicating the end of the sequence is padded to both the question and the answer. The words in vocabulary are cast into 100-dimension embeddings using GloVe (http://nlp.stanford.edu/projects/glove/ (accessed on 10 October 2020)), the embeddings of the other words of low-frequency are randomly initialized with the same scale as GloVe. The word embedding as well as the feature embedding is further trained while learning.

*Multi-document Summarization Methods*. The multi-document summarization methods introduced in Section 4.3.2 are implemented in PKUSUMSUM (https://github.com/PKULCWM/PKUSUMSUM (accessed on 10 October 2020)) [96], which is a Java toolkit supporting different summarization tasks and methods as well as integrating Stanford Tokenizer (http://nlp.stanford.edu/software/tokenizer.html (accessed on 10 October 2020)) and PorterStemmer (http://tartarus.org/~martin/PorterStemmer/ (accessed on 10 October 2020)) for text processing. We also remove the stopwords of answers based on the stopword list provided by PKUSUMSUM.

*Training Setup*. Our *ED* model is implemented using Keras (https://github.com/keras-team/keras (accessed on 10 October 2020) with Theano (https://github.com/Theano/Theano (accessed on 10 October 2020)) as backend. We use the Adam [97] optimizer with default parameters in training and beam size of 10 in decoding. Batch size is set to 128 and each epoch processes 235 batches. The model is trained on a single TITAN Xp GPU. With the best model configuration (discussed in Section 5.2), ED-SoO, ED-SoI, ED-SoC and ED-SoCF take around 22, 20, 10 and 23 h to converge, respectively. To make the training process to be repeatable and reliable, training examples are fed to models with the fixed sampling sequence.

#### 5.1.3. Automatic Evaluation Metrics

Due to the statement flexibility of questions (i.e., the same question could be asked in different ways), we incorporate three types of automatic metrics to measure the lexical similarity, human consensus and semantic similarity of the generated questions with respect to those of dataset D.

**Lexical Similarity Metrics**. The computation of lexical similarity is based on the word-overlap between generated questions and the reference. We consider the following three lexical similarity metrics:

L-B*_n_* **B**LEU [98] is a widely used precision-based metric in machine translation. It analyzes the co-occurrences of *n*-grams between the generated question and the reference. *n*-gram represents the word group with *n* words, BLEU-n score is the weighted geometric mean of the individual n-gram precision, where n=1,2,3,4.
L-R*_L_* **R**OUGE [99] is a popular recall-based metric in text summarization. We evaluate the generated questions using ROUGE-L, which is the F-measure of the longest common subsequence (LCS) between the generated question and the reference.
L-M **M**ETEOR [100] is a metric based on the harmonic mean between unigram precision and recall, which correlates better at the sentence level with human evaluation [101].

**Consensus Metric**.

C-Cr **C**IDE**r** [102] metric is firstly proposed to measure the human consensus of image captions. It computes the TF-IDF weights for each *n*-gram, and computes the CIDEr score by averaging the cosine similarity of the TF-IDF vectors of two sentences. We apply this metric to evaluate how human-like the generated questions are.

**Semantic Similarity Metrics**. This type of metric computes the semantic similarity with the help of word embeddings. Like [102], we first obtain the sentence embedding from embeddings of words making up this sentence, then calculate the cosine similarity between sentences’ embeddings as the semantic similarity. These metric scores remain high even though the generated question is stated in a different way but expresses the similar meaning, while the lexical metric scores will drop markedly.

S-ST **S**kip-**T**hought Cosine Similarity. The Skip-Thought model [103] uses an LSTM to encode the sentence into an embedding, which has a robust performance on semantic relatedness task.
S-EA **E**mbedding **A**verage Cosine Similarity. The embedding of a sentence is computed by averaging the embedding of each word of it.
S-VE **V**ector **E**xtrema Cosine Similarity. Vector Extrema [104] computes the sentence-level embedding by extracting the most extreme value of each dimension of the embeddings of the words composing this sentence.
S-GM **G**reedy **M**atching Score. Greedy Matching [105] first greedily matches the word of one sentence to the word of another sentence based on the cosine similarity of their words’ embeddings, then computes the sentence similarity by averaging these similarities.

The three types of metrics are computed using the Python toolkit published by Sharma (https://github.com/Maluuba/nlg-eval (accessed on 10 October 2020)) [101], in which the semantic metrics make use of the 300-dimension word embeddings from GloVe.

#### 5.1.4. Human Evaluation Metrics

Humans evaluate the generated question from two aspects:

*Is it well expressed?* It covers the correctness of question word, referential clarity and grammatical correctness [19].

*Can it guide teens to read?* It covers the attracting of teens’ attention, the essential message of questions for multiple articles and the highlight of teens’ stress (as introduced in Section 1.1).

### 5.2. Results

#### 5.2.1. Experiment 1: Performance Comparison of Different Solutions

We perform 4 tests in this experiment:

**Test 1.** This test makes use of two non-ED methods as the contrast experiments. Given the testing articles, both the methods take the question generation task as retrieval of the most similar question from the training dataset. The difference lies in the ranking schema for candidate questions:

- **BoW**. **B**ag-**o**f-**W**ords method represents the articles to a question as a word frequency vector and ranks the questions in the training dataset based on the cosine similarity between their articles’ vectors and that of the testing articles.
- **BM25** [106]. BM25 takes the questions in the training dataset as queries and the testing articles as a document. The query of the highest BM25 score with the document is chosen as the question of the testing articles.

**Test 2.** To investigate the feasibility of the underlying encoder-decoder model and the impact of diverse-content issue of multiple articles, we apply our basic encoder-decoder model *ED* and the state-of-the-art encoder-decoder model *ngq* to generate a question from the single article (like the traditional question generation task):

- **ED-avg**. We train *ED* on every article of training examples. While testing, we generate a question separately from each article of testing examples and take their **average** scores on evaluation metrics as the final score. Article’s lead summary is truncated to be of length m=50.
- **ngq-avg**. It is the same as ED-avg except that we substitute our encoder-decoder *ED* with *nqg* model. *nqg* [10] is the state-of-the-art model for question generation on SQuAD dataset, both its encoder and decoder are 2-layer LSTMs with 600 hidden units while the LSTM of the encoder is bidirectional. *nqg* implements two variations of encoders: one that only encodes the sentence and the other encodes both sentence-level and paragraph-level information, the first one achieves the best performance. *nqg* generates the output with global attention mechanism [107]. We use the better *nqg* model with only sentence-level encoder.

**Test 3.** We apply our solutions to generate questions from multiple *N* articles (*N* = 4). For ED-SoO, ED-SoC and ED-SoCF, the length of the lead summary of the single article is m=50. For ED-SoI, we use the coverage multi-document summarization and set the summary length as s=165. The features embedded into ED-SoCF are POS, TF-IDF, upvotes and NER.

**Test 4.** Human generation and human evaluation. We compare the machine’s behaviors with those of human users by inviting six volunteers (who are all graduate students, including 2 native English speakers and 4 non-native English speakers) to devise a question from multiple articles for the purpose of guiding reading. A total of 107 (question, answers) pairs are randomly sampled from the testing set, and are equally divided into 6 sets, one set per volunteer. Furthermore, we invite another 3 volunteers to evaluate the original questions in TeenQA, the volunteer generated questions and the model generated questions on the human evaluation metrics listed in Section 5.1.4.

Experimental results on the automatic evaluation metrics are reported in Table 8, from which we can get the following interesting observations:

(a) Two non-ED methods perform much poorer than the ED-based models. It is hard to guarantee the suitable question appears in the training dataset when the dataset is restricted by size.
(b) ED-avg and nqg-avg are also not satisfying in our question generation task, especially on the consensus metric and the semantic similarity metrics. Although both of them are feasible to generate fairly fluent questions, their content terribly digresses from the general idea, which reflects the limitations of the single encoder-decoder in handling the diverse-content articles. Among the two methods, ED-avg is worse than nqg-avg, because ED-avg uses the most basic encoder-decoder *ED* in which the word embedding dimension and the size of LSTM units is much smaller, the encoder is unidirectional and the optimizer is set to Adam with the default learning rate and parameters without intensively study. Even with this weak encoder-decoder, our solutions based on *ED* in Test 3 still beat nqg-avg by a large margin, demonstrating the effectiveness of our solutions.
(c) ED-SoO, ED-SoI and ED-SoC comprehensively improves the question quality but contributes most to the consensus metric and the semantic similarity metrics. It reveals the ability of our solutions to capture the key information across articles. Among the three solutions, ED-SoC achieves the best performance, indicating that summarization on articles’ vector representations with hierarchical attention is the best synthesizing method for multiple articles.
(d) Features can greatly enhance the performance on all metrics. After adding POS, TF-IDF, upvotes and NER features, the ED-SoCF model increases the lexical scores and consensus score by more than 30% and 60%, respectively compared to nqg-avg. Finally, ED-SoCF is 27%, 18% and 13% higher than ED-SoO, ED-SoI and ED-SoC on lexical metrics, 48%, 53% and 38% higher on consensus metric. On some semantic scores (e.g., Embedding Average Cosine Similarity), ED-SoCF is comparable with that of volunteers (87.2 vs. 87.7).
(e) Humans do very well on the consensus metric and the semantic similarity metrics, while they perform not that good on the lexical similarity metrics. This is quite different from previous text summarization tasks and question generation task where the humans can achieve very high scores on BLEU and ROUGE. The reason is that, even though humans can capture the key information of the articles, it is still hard to express the question in the same form as the original one due to the statement flexibility of natural language.

Human evaluation results are reported in Table 9. We find the questions generated by volunteers are very comparable with original ones in TeenQA, verifying the rationality to use the human results as the upper bound of the task in Table 8. In addition, ED-SoCF could not only generate a fairly valid question (especially in question word and referential clarity), but it also performs well in guiding reading. Yet, the grammatical correctness and essential message need to be further improved in future work.

**Two Examples**. Table 10 provides an example for concrete study of the performance of each solution. To get an idea of the difficulty of our task, we suggest readers try to derive the question by hand at first.

At the first glance, each article seems talking about different things, but after reading all of them, humans can conclude that the question should be of methodological type and the key information is about *spiritual journey*. It should be noted that the question generated by the volunteer begins with *how to* while the original one begins with *what is the best way*, suchlike differences in expression habits can explain volunteers’ unsatisfactory performance on the lexical similarity metrics in one aspect.

Looking at the results of the basic *ED* model. It generates one question respectively from each article. Without considering the overall information, the generated questions are all biased towards the individuality of each article, some of them are far away from the main idea.

ED-SoO picks the best one (the second one) from the questions generated by *ED*. It is much better but the word *monk* is not that suitable.

As for ED-SoI, it abstracts out the phrase *develop a spiritual*, which is not present in articles but fairly close to the main idea. However the question words *how long* are inaccurate.

Finally, let us look at the question generated by ED-SoC and ED-SoCF. They both capture the correct question words *what is the best way*. Further on, with the help of features, ED-SoCF generates a more complete sentence and conveys almost the same main idea with the original one.

**Figure 11:**
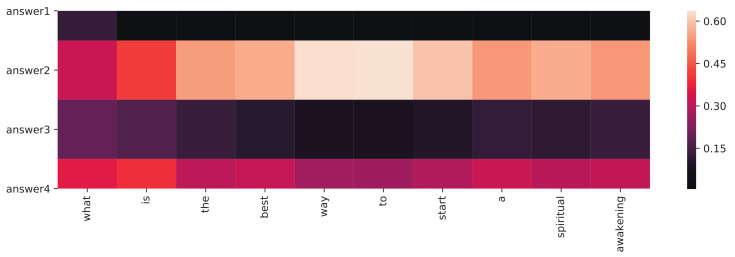
Article attention visualization.

Figure 11 explains how multiple articles are summarized in ED-SoC/F. The decoder gathers context from four inputs at each inference step, and the grids indicate the attention weights ($\alpha_{i}^{t}$). As the figure shows, answer2 and answer4 contribute more context for the generated question since they both contain more description about the theme *spiritual awakening*.

On the other hand, Table 11 gives another example showing the potential hardness of our task. The theme of this example is how to prepare for an exam. Our model successfully gets the general idea but unfortunately misses the correct exam name: it should be *SBI* rather than *IBPS*. The reason is: the model has learned to pay more attention to the first two answers through the hierarchical attention mechanism owing to their higher quality, but it is difficult to figure out the correct exam name from the first 2 answers because *SBI* and *IBPS* appear together in the text. The only clue to get the right exam name is in Answer3, however Answer3 is too poor an answer to get much attention. Thus, beyond the hierarchical attention, designing a new feature to distinguish key words from the redundant text should be helpful in our task.

#### 5.2.2. Experiment 2: Impact of Different Features in ED-SoCF

For the best ED-SoCF solution, we look into the performance gains of different features. We conduct 5 tests, in which the number of features varies from 1 to 5. In each test, we add the feature bringing forth the biggest performance gain, then based on the new feature combination we do the next test until all features are added.

This method of feature selection can not guarantee the optimal feature combination is obtained, but can get a relatively optimal solution with the complexity of feature selection decreasing from $O(2^{n})$ to $O(\frac{n(n+1)}{2})$.

From Table 12, we can see that:

(a) Any of the features could improve the performance, of which upvotes mainly contributes to semantic similarity metrics; TF-IDF mainly contributes to lexical similarity metrics (especially BLEU of long grams) and consensus metric; topic, NER and POS contributes to all metrics, but individually speaking, POS is the best feature.
(b) The optimal combination of features is [POS, TF-IDF, upvotes, NER]. To our surprise, the combination of best single features (e.g., [POS, NER]) does not necessarily get a better result, and integrating all features obtains a performance even worse than the single feature. The reason might be two-fold: (1) adding new feature(s), the dimension of the input increases, so the model is harder to converge at the optimal point with the same training parameters and the fixed size of training data; (2) there might be much overlapping effects between new feature(s) and the existing features, thus adding more features can not bring in much performance gain.

As a result, ED-SoCF takes 4 features: POS, TF-IDF, upvotes and NER.

#### 5.2.3. Experiment 3: Impact of Articles’ Summary Lengths

This experiment aims to find the best length *m* of article’s lead summary, and the best length *s* of articles’ multi-document summary. While testing *m*, without impact on comparison result, we train our basic encoder-decoder model *ED* only on the first article of each training example and vary the length of the lead summary to 50, 165 and 200. While testing *s*, without loss of generality, we take the Submodular method as an example to analyze the impact of different summary length (50, 165, 200) on performance of the ED-SoI model. The result is shown in Table 13.

It is observed that the information quantity and density of the input significantly influence the performance. Increasing the summary length from 50 to 165, the information quantity is improved, the model performance is also improved at the same time. If continuing increasing the summary length to 200 words, the performance dramatically drops because the information density lowers down and too much subjective information confuses the model and hurt the performance. Therefore, we fix the length of multi-document summary to be 165 words. In the meantime, we note that increasing the input length of *ED* from 50 to 165 does not gain many benefits but costs much longer time for model to converge. Therefore, we still use the first 50 words as the article’s lead summary.

Finally, we set m=50 and s=165.

#### 5.2.4. Experiment 4: Impact of Multi-Document Summarization Methods in ED-SoI

This experiment tests the effect of different multi-document summaries on model performance. Based on the result of experiments in Section 5.2.3, we fix the multi-document summary to be of the best length s=165 and change the summarization method to be Coverage, Centroid, Textrank, ILP, ClusterCMRW and Submodular.

As Table 14 shows, multi-document summary can greatly enhance the model, but the performance of different summarization methods differs a lot.

(a) In the view of information density. Combined with Table 10, it is interesting to find that the performance is highly related to the information density (i.e., words overlap between multi-document summary and the question) of the input.
(b) In the view of information generality. The baseline method coverage performs best, proving our assumption that the generic information mostly spreads in the lead sentences. Oppositely, Centriod, TextRank and ILP which take into account all sentences in articles are affected by the subjective information and perform even poorer than the basic solution ED-avg. Cluster is better than the above 3 methods for reason that it collects sentences based on detected themes rather than weights of single sentences. Submodular is the best multi-document summarization method in the work of Zhang et al. in DUC 2004 (http://duc.nist.gov/duc2004 (accessed on 10 October 2020)) [96], but it falls behind the coverage method by a large margin in our task, indicating the difference of articles between TeenQA and DUC 2004 (newspaper news).

Consequently, we choose coverage in ED-SoI.

## 6. Conclusions

In this paper, we proposed to generate an instructive question from the multiple recommended articles to guide teens to read for the sake of good reading experiences and effective E-bibliotherapy. For model training and testing, we collected and built a novel large-scale QA dataset TeenQA, and analyzed its feasibility for our task. Our extensive experimental results showed the proposed ED-SoCF model surpassed the traditional non-ED methods and the previous state-of-the-art model *nqg* on SQuAD by a large margin, demonstrating the ability of our solution on generating good instructive questions for guiding reading.

The encoder and decoder used in our work can be further enhanced by more advanced mechanisms (like copy mechanism and pointer generator) and more advanced encoder/decoder variants (like Transformer [108] and Transformer-XL [109]). Although all of them are helpful, they are beyond the scope of this paper. The paper aims to set forth a new task and the corresponding solutions, which can be easily extended to other application domains. For example, TeenQA and the methodology of ED-SoCF can be migrated to all scenarios where a short natural language sequence is required to be generated from multiple long inputs, like general question generation tasks for reading comprehension and headline generation tasks for multiple documents.

On the other hand, there exists the potential overfitting problem with any specific dataset. In this case, building meta-learning models that can adapt to new datasets is also worthy of exploration. Here, meta-learning aims to learn the common parts of different meta-tasks, such as how to extract important features and compare samples’ similarity, and forget the task-specific parts, so that the model can still work effectively on a new dataset.

## Figures and Tables

**Table 1:** An article in *TeenRead*.

**Article Title**	“Advice for Career Planning”
**Stress Category**	career planning
**Article Content**	A career plan is not unlike a battle plan: Both have two dimensions, tactical and strategic plans, for winning the battles and eventually the “war”. Fortunately, planning a career is a much happier prospect than having to wage a war. The similar requirements for short-term, tactical plans and long-term strategic goals and vision make the comparison valid.
	When you are mapping out the future of your career and your expected career development, it is important to have clear and well thought-out plans of both types, for success. Although career planning, unlike a real battle, is a highly personal process, as it involves your personal aspirations and motivations, it is often a good idea to get some outside input from a professional job coach or a counselor in your industry of choice.

**Table 2:** Recently released QA datasets.

Dataset	Source	Context	Question	Answer
SQuAD	Wikipedia	passage	human generated	span of words
NewsQA	CNN news	document	human generated	span of words
MCtest	children’s stories	story	human generated	multiple choices
Simple Question	Freebase	subject-relation-object	human generated	object
MARCO	Bing	passage	Bing queries	human generated
WikiQA	Bing	sentences	Bing queries	sentence selection
TriviaQA	trivia/Bing	documents	trivia enthusiast	span of words
rc-data	CNN/DM news	document	cloze	entity
TeenQA	Quora	–	human generated	human generated

**Table 3:** Question topic seeds in TeenQA.

Stress Category	Question Topic Seeds
Study	Studying, Study-Habits, Study-Strategies, Colleges-and-Universities, College-and-
	University-Admissions, Education, Higher-Education, Educational-Courses, Educational-
	Resources, Competition, Contests-and-Competitions, Grades-education, Academic-Degrees,
	Homework, Exams-and-Tests, Exam-Strategies, Recommendations, College-Advice,
	The-College-and-University-Experience, Graduations, Graduate-School-Education,
	Postgraduate-Education, Scientific-Research, Learning, Studying-Abroad
Family	Family, Family-Relationships-and-Dynamics, Interpersonal-Interaction-with-Family-
	Members, Parents, Siblings, Dating-and-Relationships-1, Marriage
Romantic Relation	Affection, Love, Frustration, Dating-Advice, Psychology
Peer Relation	Interpersonal-Interaction, Social-Advice, Friendship, Roommates, Classmates,
	Social-Psychology
Self-Cognition	Self-Awareness, Self-Motivation, Self-Improvement, Cognitive-Psychology
Employment	Employment, Self-Employment, Jobs-and-Careers, Job-Interviews, Job-Interview-
	Questions, Job-Searches, Internships, Part-Time-Jobs, Internship-Hiring, Hiring, Career-
	Advice, Personal-Finance
Life	Life-and-Living, Tips-and-Hacks-for-Everyday-Life, Life-Lessons, Life-Advice, Motivation,
	Psychology-of-Everyday-Life

**Table 4:** An example of QA pairs in TeenQA.

Field	Content
question	How do top students study?
category	Study
topic	Study-Strategies
follows	19948
answer1	I will speak on behalf of a close friend of mine, who attended an unknown university from where I am from (Lima, Peru), and got accepted for a fully funded PhD to work with the world-leaders (including Nobel Laureates) at the Systems Biology and Computational Biology at Harvard…
upvotes1	9622
answerer1	Arturo Deza
description1	Robot Opthalmologist
date1	26 October 2016
answer2	I am an above-average student at Caltech. I do not think I study particularly hard, but I do: 1—Get 8–9 h of sleep a night. This allows me to go to class well-rested and do my problem sets with greater efficiency. 2—Always go to class. Even if the lectures are not…
upvotes2	6306
answerer2	Jessica Su
description2	CS PhD student at Stanford
date2	9 October 2012
answer3	What is my story: I went to IIT, one of the best colleges in India, and stood first in my class. Thereafter I did a PhD in Chemical Engineering, and worked hard towards my studies. I put a lot of hours towards my studies in my life, and I have seen some other students whowere far…
upvotes3	5491
answerer3	Rohit Malshe
description3	studied at Doctor of Philosophy Degrees
date3	28 March 2017
answer4	I was a decent student back when I was an MIT undergrad and now that I am a prof, and I really want my students to do well. Every semester, I email them a link to the following article by Cal Newport: http://calnewport.com/blog/2007/ (accessed on 10 October 2020)…, which pretty much summarizes everything in…
upvotes4	3740
answerer4	Ben Leong
description4	Associate Professor of Computer Science
date4	24 May 2012

**Table 5:** Distribution of stress categories in TeenQA.

Stress Category	#Question	Percentage
Study	220,819	31.7%
Employment	187,949	27.0%
Life	65,312	9.4%
Family	57,328	8.2%
Romantic Relation	56,005	8.0%
Peer Relation	55,407	7.9%
Self-Cognition	54,285	7.8%
Total	697,105	1

**Table 6:** Number of answers’ *Upvotes*.

#Upvotes	#Answer 1	#Answer 2	#Answer 3	#Answer 4	Percentage
[0,5)	424,473	418,566	342,121	270,178	74.1%
[5,10)	95,827	53,667	33,783	23,574	10.6%
[10,40)	88,207	43,177	27,403	20,138	9.1%
[40,+)	65,630	27,122	16,866	12,163	6.2%
All	674,137	542,532	420,173	326,053	1

**Table 7:** Distribution of question types in TeenQA.

Question Type	Percentage	Example
Factoid	18.4%	Where can I publish my article for free?
List	11.4%	What are the easiest musical instruments to learn to play well?
Hypothetical	4.1%	What should I do if my phd advisor does not answer my Emails?
Confirmation	19.5%	Is Fedora better than Ubuntu for Software Development?
Causal and Explanatory	19.5%	Why do the most important lessons in life hurt so much to learn?
Methodological	27.1%	How could I get rich as a teenager?

**Table 8:** Performance on automatic evaluation metrics.

Model	Lexical Similarity						Consensus	Semantic Similarity			
	L-B${}_{1}$	L-B${}_{2}$	L-B${}_{3}$	L-B${}_{4}$	L-R${}_{L}$	L-M	C-Cr	S-ST	S-EA	S-VE	S-GM
BoW	19.1	10.1	6.2	4.1	18.1	9.9	45.1	45.4	81.7	45.0	62.6
BM25	13.8	6.2	3.2	1.8	15.2	8.0	37.4	44.5	82.1	45.6	63.3
ED-avg	23.6	13.6	8.8	6.0	23.4	11.7	59.3	47.2	84.2	49.4	66.4
nqg-avg	23.9	14.9	10.3	7.5	24.2	12.1	68.1	47.7	83.6	49.5	66.7
ED-SoO	24.4	14.7	9.9	7.1	25.2	12.5	74.2	48.2	84.1	50.3	67.1
ED-SoI	27.4	16.0	10.2	6.9	27.2	13.2	71.6	49.1	85.8	52.4	68.6
ED-SoC	28.7	16.8	10.8	7.3	27.8	14.0	79.4	48.8	86.3	53.8	69.8
ED-SoCF	**32.3**	**20.4**	**14.2**	**10.3**	**32.0**	**16.1**	**109.5**	**50.8**	**87.2**	**55.7**	**71.3**
↑	*35.1%*	*36.9%*	*37.9%*	*37.3%*	*32.2%*	*33.1%*	*60.8%*	*6.5%*	*4.3%*	*12.5%*	*6.9%*
Volunteers	*40.6*	*28.2*	*19.8*	*13.9*	*39.8*	*21.8*	*180.9*	*59.4*	*87.7*	*65.8*	*76.5*

The metrics from left to right are **B**LEU-**1**, **B**LEU-**2**, **B**LEU-**3**, **B**LEU-**4**, **R**OUGE-**L**, **M**ETEOR, **CIDEr**, and scores of **S**kip-**T**hought, **E**mbedding **A**verage, **V**ector **E**xtrema and **G**reedy **M**atching. The highest score of our solutions on each metric marked in **bold**. The volunteers’ scores are shown in *italics*. ↑ indicates the performance gains of the best solution ED-SoCF compared to the state-of-the-art model nqg-avg.

**Table 9:** Performance on human evaluation metrics.

Questions	Is the Question Well Expressed?			Can the Question Guide Teens to Read?		
	Question	Referential	Grammatical	Attracting	Essential	Stress
	Word	Clarity	Correctness	Attention	Message	Highlight
Original	5	5	4.6	4.8	4.6	3.9
Volunteers	4.7	4.7	4.7	4.6	4.4	4.4
ED-SoCF	4.5	4.6	3.2	4.3	3.5	3.3

The scores are from 0 to 5, indicating the quality from worst to best.

**Table 10:** An example for concrete study.

Original	*What is the best way to start a journey in spirituality?*
Volunteers	How **to start a spiritual journey**?
ED	What is the point of living in India?
	How can I become a spiritual monk?
	Am I too old to start learning martial arts?
	What is the best way to spiritual enlightenment?
ED-SoO	How can I become **a spiritual** monk?
ED-SoI	How long does it take **to** develop **a spiritual**?
ED-SoC	**What is the best way to** become **a spiritual**?
ED-SoCF	**What is the best way to start a spiritual** awakening?
answer1	The truth is that the destination is here right now, yet we always think in terms of a journey. This means, if we think a journey is involved, then we put the goal or destination far away from us. Let me give a small tip based off of a lifetime of practice, of many ups and downs, a lot of study, a lot of meditation—traveling all over the planet: Sit down when you have a half an hour. Do not be in a rush. Bring your awareness to your heart. Make the thought, “My heart is my destination. I am with my goal.” Allow yourself to feel this for some time. Always start your practice, whatever it will be, with this thought that you are already there. This will set the tone for your journey-less journey. This is the correct attitude to adopt with great confidence—because it is the truth. May your arrival precede your departure. Love to all, Brian.
answer2	Become part of spiritual organization like ISKCON, attend their weekly programs on various spiritual topics. Start reading spiritual books written by enlightened masters like Srila Prabhupada, spend time with spiritual people. The ISKCON devotees, do more of spiritual activities like chanting, have Deity Darshan, honour Krishna Prasadam, attend festivals, follow a spiritual lifestyle and you would have begun your spiritual journey.
answer3	I would suggest that you go and do the entry level course of THE ART OF LIVING, which is YES+! (youth empowerment and skill workshop) for the age group of 18–35. or HAPPINESS PROGRAM (also known as WAVES OF HAPPINESS) for age group of 18+, for your spiritual journey you will learn an excellent technique called SUDARSHAN KRIYA, which will harmonize your body and soul with the nature. Also, along with it, you will get some knowledge point in the course, which will be beneficial to you for living happy life in this materialistic world. I hope this will help you. Stay happy stay blessed.
answer4	Asking this question tells me you are already on your spiritual journey. Spiritual energies now are very strong and many people are responding to higher energy frequencies. Asking for the best way makes a lot of sense. One can walk around the mountain for several life times before reaching the higher spheres or one can do the same in one life time going up straight step by step…

In the generated questions, we highlight the parts appearing in the original question in bold. The table only displays the first few sentences of answers limited to space.

**Table 11:** An example showing the hardness of our task.

Original	*How should I start my preparation for sbi po?*
Volunteers	How do I prepare for sbi po?
ED-SoCF	How do I prepare for the ibps po?
answer1	As you are starting your preparation, I would suggest in the 1st week to solve 2–3 previous year prelim papers of SBI PO or IBPS PO exams and then analyze it deeply. See which topics do you know and which topics you do not. Which topics took more time and which topics less, simultaneously you can analyze it on various parameters. Once analysis is done, make a proper study plan by giving extra time to your weakness and regularly revising it so you can make it your strength. Suggestion: —If you want help in your preparation you can subscribe to my YouTube channel for regular update and tips to crack bank exams. 1—Prepare 10–12 daily by giving 2–3 h to each subject. 2—Attempt one mock daily for prelim and analysis. 3—Give time to weak topics…
answer2	Your first move should be: 1—Join a good institute for the preparation like crash course. 2—Never miss a class. A regular is the most important thing you have to do. 3—Learn tricks and concepts to solve questions. 4—Solve all previous year papers. 5—Take up a topic daily and clear your concept. 6—Give as much MOCK TESTS as you can. 7—Practice questions daily. 8—For English, you should learn the techniques of solving questions. You can also download an app to practice thousands of questions I can suggest one such app LearnAir SSC BANK RAILWAYS-Android Apps on Google Play LearnAir is an app specially designed for the aspirants preparing for exams like SSC CGL, IBPS, CHSL, MTS, SBI, RRB, AND ALL GOVERNMENT AND BANKING EXAMS. This app includes…
answer3	As per the SBI merger this year, there will be no vacancies next year for SBI PO. Therefore, my advice is to focus on other exams such as SSB, SSC, etc. To get your job. This is an exclusive information by SBI.
answer4	Read Avi Singh’s answer to How do I prepare for SBI/IBPS PO?

**Table 12:** Influence of features on ED-SoCF.

	Lexical Similarity						Consensus	Semantic Similarity			
	L-B${}_{1}$	L-B${}_{2}$	L-B${}_{3}$	L-B${}_{4}$	L-R${}_{L}$	L-M	C-Cr	S-ST	S-EA	S-VE	S-GM
ED-SoC	28.7	16.8	10.8	7.3	27.8	14.0	79.4	48.8	86.3	53.8	69.8
**Feature(s)**											
+U (upvotes)	29.3	17.0	10.8	7.3	28.3	14.1	79.8	49.0	86.5	53.9	69.8
+TI (tf-idf)	28.9	17.5	11.6	8.0	28.5	14.0	81.5	48.9	86.2	53.1	69.5
+T (topic)	29.9	17.6	11.5	7.9	28.9	14.4	84.0	48.9	86.5	54.0	70.1
+N (ner)	30.4	18.3	12.2	8.4	29.5	14.6	87.7	49.8	86.7	54.5	70.3
+P (pos) *	30.9	18.7	12.8	9.2	30.3	15.2	95.7	50.2	86.7	54.8	70.6
+[P,U]	30.3	18.2	12.2	8.5	29.3	14.6	87.6	49.8	86.6	53.9	70.1
+[P,T]	30.2	18.5	12.4	8.7	29.4	14.6	89.6	49.8	86.4	53.5	69.7
+[P,N]	30.6	18.8	12.6	8.9	29.9	14.9	92.9	49.5	86.6	54.0	70.2
+[P,TI] *	31.8	20.1	13.9	10.1	31.5	15.4	105.0	50.5	86.9	55.1	70.9
+[P,TI,T]	29.6	18.1	12.3	8.8	28.7	14.3	88.9	49.2	86.4	53.7	69.7
+[P,TI,N]	30.9	19.0	12.9	9.1	30.1	14.8	91.9	49.8	86.5	53.8	70.1
+[P,TI,U] *	31.2	19.2	12.9	8.9	30.4	15.2	93.7	50.1	86.9	54.8	70.4
+[P,TI,U,T]	31.5	19.9	13.7	9.9	30.8	15.2	101.0	50.6	86.7	54.4	70.5
+[P,TI,U,N] *	**32.3**	**20.4**	**14.2**	**10.3**	**32.0**	**16.1**	**109.5**	**50.8**	**87.2**	**55.7**	**71.3**
+[P,TI,U,N,T] *	29.9	18.1	12.3	8.7	29.3	14.3	88.5	49.3	86.4	53.8	69.8

In order to reduce the random error, we repeat each test 3 times to get the average results. The best combination of feature(s) in each group is marked with *. The highest score on each metric is marked in **bold**.

**Table 13:** Influence of summary length.

Model	Lexical Similarity						Consensus	Semantic Similarity			
	L-B${}_{1}$	L-B${}_{2}$	L-B${}_{3}$	L-B${}_{4}$	L-R${}_{L}$	L-M	C-Cr	S-ST	S-EA	S-VE	S-GM
ED-50	21.1	11.2	6.9	4.5	20.7	10.0	41.7	**45.4**	83.2	46.6	64.9
ED-165	**21.5**	**11.6**	**7.4**	**5.2**	**21.1**	**10.3**	**47.0**	45.3	**83.7**	**47.3**	**65.3**
ED-200	18.8	9.8	6.1	4.0	18.7	8.6	30.6	43.8	82.0	43.2	62.8
ED-SoI-50	23.0	12.6	7.7	4.9	22.4	11.0	56.0	46.8	84.4	49.6	66.1
ED-SoI-165	**25.3**	**14.3**	**9.2**	**6.2**	**24.8**	**12.3**	**64.3**	**48.4**	**85.0**	**50.9**	**67.6**
ED-SoI-200	22.7	12.5	7.9	5.2	22.0	11.0	51.9	46.9	84.0	48.2	65.7

The highest score on each metric in each group is marked in **bold**.

**Table 14:** Influence of multi-document summarization method on ED-SoI.

Summary	Lexical Similarity						Consensus	Semantic Similarity			
	L-B${}_{1}$	L-B${}_{2}$	L-B${}_{3}$	L-B${}_{4}$	L-R${}_{L}$	L-M	C-Cr	S-ST	S-EA	S-VE	S-GM
centroid	22.0	11.8	7.4	4.9	21.3	10.4	47.3	46.2	84.0	48.1	65.6
ilp	22.2	12.2	7.6	4.9	21.8	10.7	49.3	46.5	84.1	48.5	65.8
textrank	22.5	11.9	7.2	4.7	21.9	11.0	54.0	47.2	84.4	49.5	66.2
clustercmrw	25.2	13.7	8.5	5.4	24.3	12.4	64.4	48.4	85.1	51.8	67.7
submodular	25.3	14.3	9.2	6.2	24.8	12.3	64.3	48.4	85.0	50.9	67.6
coverage	**27.4**	**16.0**	**10.2**	**6.9**	**27.2**	**13.2**	**71.6**	**49.1**	**85.8**	**52.4**	**68.6**

The highest score on each metric is marked in **bold**.

## Data Availability

The data presented in this study are openly available in https://github.com/xinyx/TeenQA (accessed on 6 May 2021).
